# Supplementary material for: Neurochemical Characterization of Neurons Expressing Estrogen Receptor β in the Hypothalamic Nuclei of Rats Using in Situ Hybridization and Immunofluorescence
Source: Int J Mol Sci. 2019 Dec 23;21(1):115. doi: 10.3390/ijms21010115 (PMC6981915; doi:10.3390/ijms21010115)
Supplement: Supplementary file 1 [file ijms-21-00115-s001.zip › Supplemental figures (Kanaya et al)/Supplemental figure legends (Kanaya et al).docx]

**Supplemental figure legends**

**Supplemental Fig. 1.** Distribution of moderate *Esr2* signals by RNAscope. Nissl-stained images (1-3) and schematic sagittal brain on the left side show the rostro-caudal levels in the organum vasculosum laminae terminalis (OVLT), ventrolateral hypothalamic tract (vlh), and ventrolateral parts of ventromedial hypothalamus (VMHvl) (**A**). Representative images (**B**) show moderate *Esr2* mRNA signals by RNAscope in the OVLT, vlh, and VMHvl of female rat. Scale bars indicate 2 mm in A and 200 μm in the low magnification images and 100 μm in the high magnification in B.

**Supplemental Fig. 2.** Positive and negative controls for RNAscope *in situ* hybridization (ISH) and conventional ISH of *Esr2*. Representative images show *Esr2* signals by RNAscope in ovarian granulosa cells (**A**) and in prostate epithelial cells (**B**). Magnified images in boxed area are shown to the right of the corresponding low magnification. In A and B panels, scale bars indicate 500 μm in the low magnification and 100 μm in the high magnification. Representative images show no signals of *Esr2* in the negative controls: RNase treatment before hybridization (**C**), omission of *Esr2* probe (**D**), *DapB* probe (**E**) and a DIG-RNA *Esr2* sense probe (**F**). No RNAscope *Esr2* signal was observed in the suprachiasmatic nucleus (SCN) (**G**) and corpus striatum (CP) (**H**). Scale bars indicate 200 μm in C–H. (**I**) Neuropeptide-immunoreactive cells co-expressing more than 3 particles of *Esr2* mRNA were defined as “co-expression”. Scale bar indicate 50 μm.

**Supplemental Fig. 3.** The percentage of cells containing 3–4 signal particles/cell and ≥5 signal particles/cell in neuropeptide-ir cells co-expressing *Esr2*. The percentage of cells containing 3–4 signal particles/cell and ≥5 signal particles/cell within *Esr2*-expressing kisspeptin neurons in the anteroventral periventricular nucleus (AVPV) and arcuate nucleus (ARC) **(A)**, *Esr2*-expressing arginine vasopressin (AVP) neurons **(B)**, and *Esr2*-expressing oxytocin (OXT) neurons **(C)** in the paraventricular nucleus of the hypothalamus (PVN) and supraoptic nucleus (SON).
