# Supplementary figures and images for: Neurochemical Characterization of Neurons Expressing Estrogen Receptor β in the Hypothalamic Nuclei of Rats Using in Situ Hybridization and Immunofluorescence
Source: Int J Mol Sci. 2019 Dec 23;21(1):115. doi: 10.3390/ijms21010115 (PMC6981915; doi:10.3390/ijms21010115)

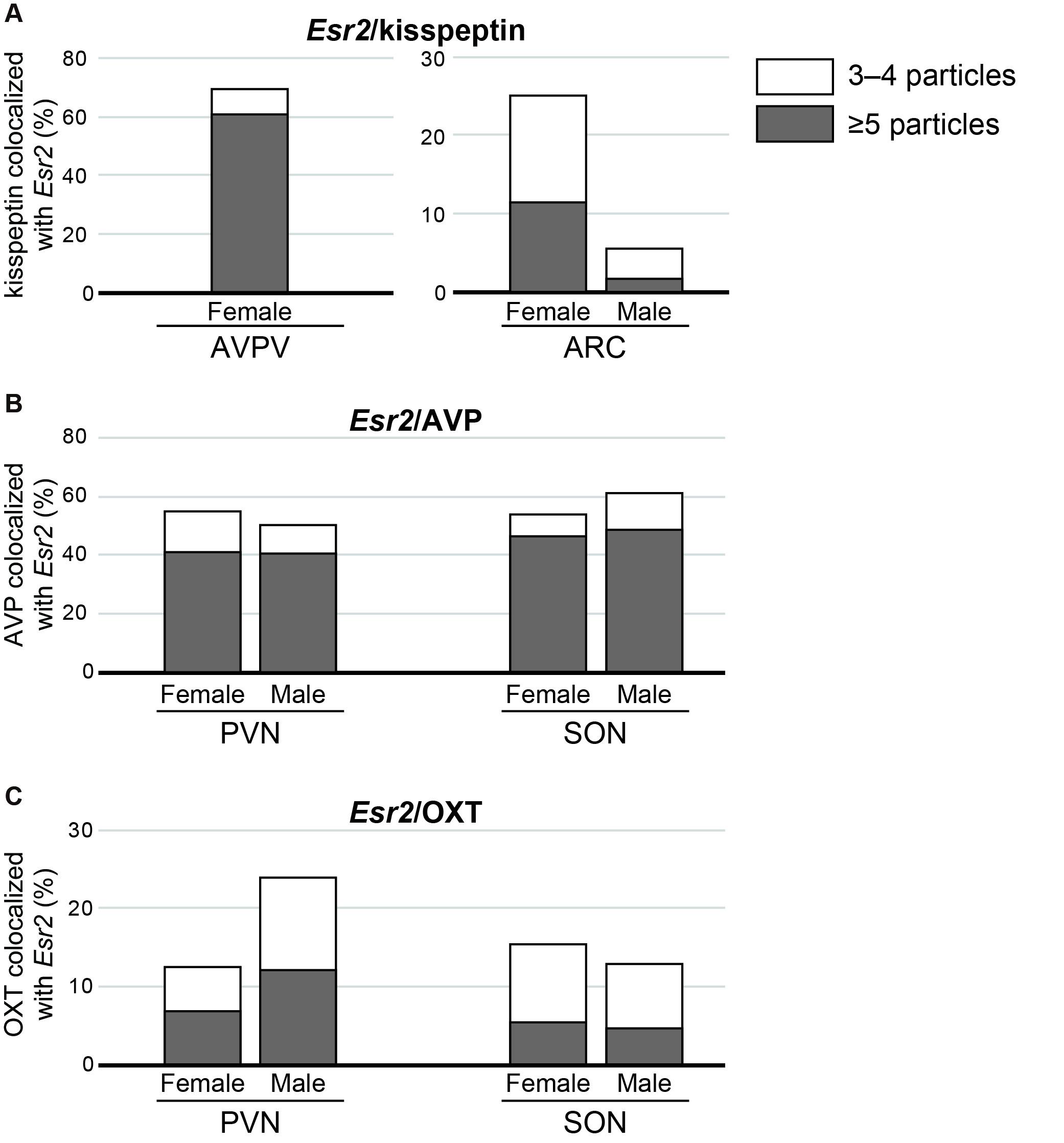

Supplement: Supplementary file 1 [file ijms-21-00115-s001.zip › Supplemental figures (Kanaya et al)/Supplemental figure 3 (Kanaya et al).jpg]

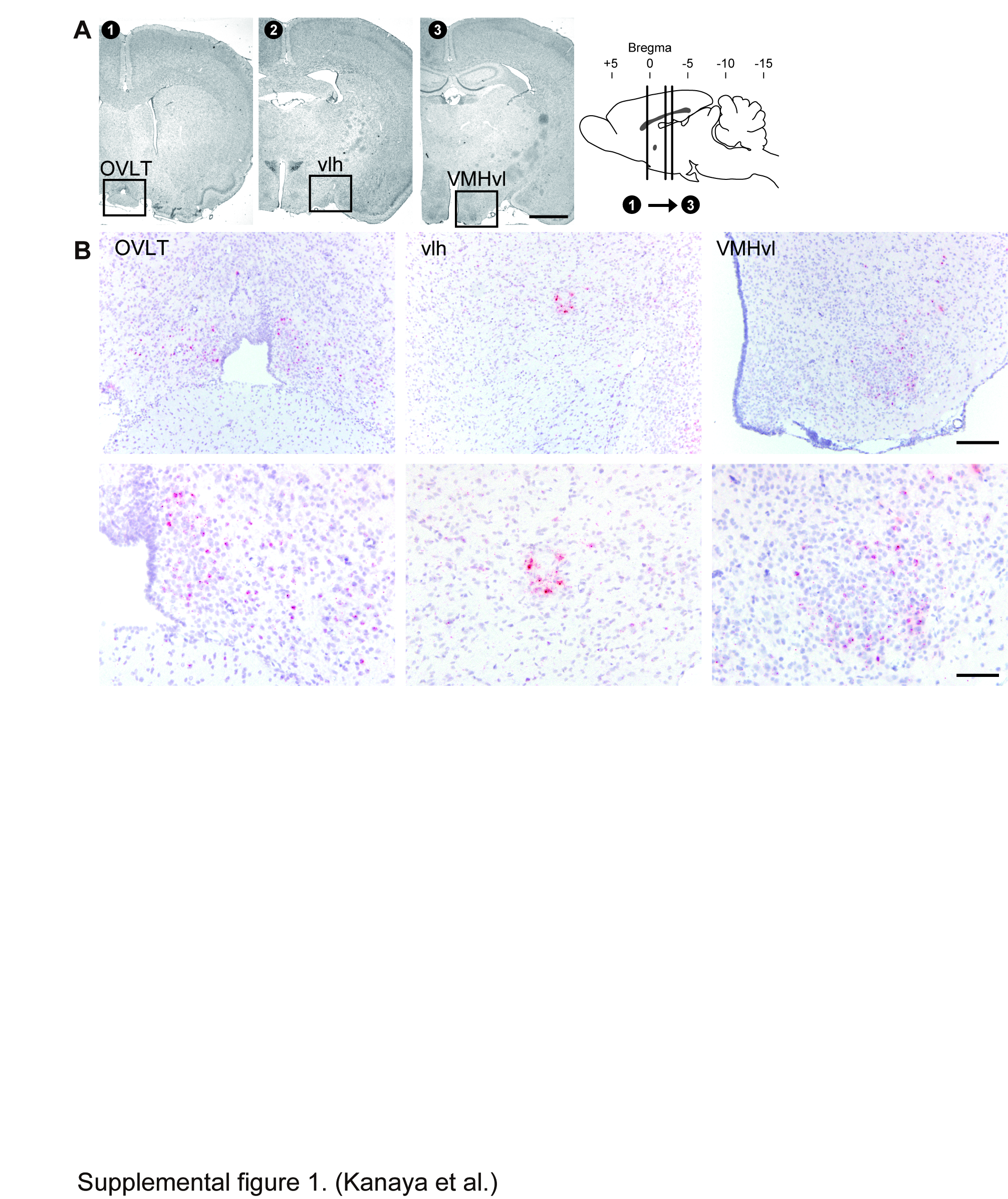

Supplement: Supplementary file 1 [file ijms-21-00115-s001.zip › Supplemental figures (Kanaya et al)/Supplemental figure 1 (Kanaya et al).tif]

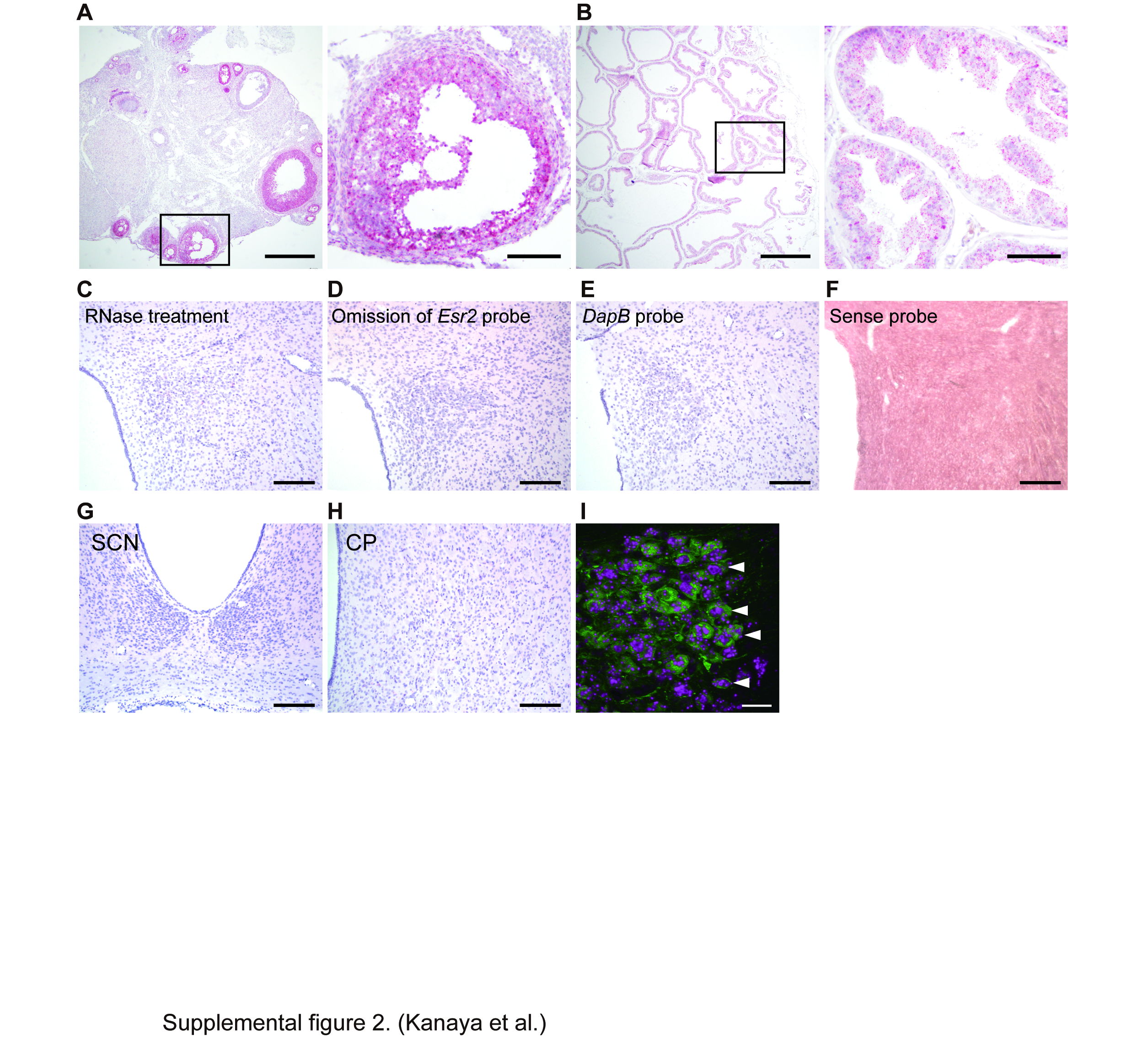

Supplement: Supplementary file 1 [file ijms-21-00115-s001.zip › Supplemental figures (Kanaya et al)/Supplemental figure 2 (Kanaya et al).tif]
